# Assessment of Renal Function in Head and Neck Cancer Patients Treated with Cisplatin: Different Biomarkers and Acute Kidney Injury Classifications

**DOI:** 10.3390/ijms24010141

**Published:** 2022-12-21

**Authors:** Nadine de Godoy Torso, Marília Berlofa Visacri, Julia Coelho França Quintanilha, Maria Aparecida Cursino, Eder de Carvalho Pincinato, Patricia Moriel

**Affiliations:** 1 School of Medical Sciences, University of Campinas, Campinas 13083-894, Brazil; 2 Faculty of Pharmaceutical Sciences, University of São Paulo, São Paulo 05508-000, Brazil; 3 Faculty of Pharmaceutical Sciences, University of Campinas, Campinas 13083-970, Brazil

**Keywords:** cisplatin, drug-related side effects and adverse reactions, acute kidney injury

## Abstract

Cisplatin is associated with dose-limiting nephrotoxicity, and the timely detection of acute kidney injury (AKI) can affect morbimortality. Therefore, this study aimed to investigate the tools for monitoring renal function in AKI. This was a retrospective, cohort study. Cisplatin-treated patients with head and neck cancer were included. Nephrotoxicity was assessed using serum creatinine, estimated creatinine clearance, serum electrolytic alterations, and plasma kidney injury molecule-1 (KIM-1). The toxicity severity was classified according to Common Terminology Criteria for Adverse Events (CTCAE), and AKI was classified by Risk, Injury, Failure, Loss, and End-stage kidney disease (RIFLE) and Acute Kidney Injury Network (AKIN). A total of 81 participants were included, of whom only 32 did not have AKI. Almost 90% of participants had a decreased estimated glomerular filtration rate five (D5) days after chemotherapy. The AKI estimate differs between AKIN and RIFLE; more participants were diagnosed by the RIFLE at D5, 19.5% versus 2.4% by AKIN, and fifteen had a discordance between these classifications. All laboratory markers showed significant changes on D5. KIM-1 appeared a possible biomarker when considering CTCAE or AKIN classifications (*p* < 0.05 on D5), but not when RIFLE classification was used (*p* = 0.0780). Further studies may seek to understand the profiles of different biomarkers together.

## 1. Introduction

Almost 800,000 new cases of head and neck squamous cell carcinoma (SCC) are reported annually [1]. Locally advanced disease (stages III and IV) represents the majority of cases, with current treatment strategies for these patients consisting of high-dose cisplatin concomitant with radiotherapy [2,3]. Despite its efficacy, cisplatin treatment is often associated with severe adverse effects, mainly as a consequence of its cytotoxic effects on healthy tissue cells. Nephrotoxicity is of particular significance; even with the accompanied use of diuretics and pre-hydration [4,5], nephrotoxicity is still a dose-limiting adverse effect [6]. Moreover, nephrotoxicity leads to a progressive decline in renal function in approximately 20–30% of patients [7]. Along with electrolyte disturbances and reduced renal filtration capacity [5], acute kidney injury (AKI) [8] is one of the main clinical manifestations of cisplatin-induced nephrotoxicity.

Thus, owing to its high prevalence and impact on treatment follow-up, it is essential to recognize nephrotoxicity for early intervention. There are currently three proposals for defining and classifying the severity of AKI: the Risk, Injury, Failure, Loss, and End-stage kidney disease (RIFLE) [9] and the Acute Kidney Injury Network (AKIN) [10]; both consider changes in serum creatinine or urinary output and the estimated glomerular filtration rate (eGFR). The third staging system was proposed by Kidney Disease: Improving Global Outcomes (KDIGO) and represents the combination of RIFLE and AKIN criteria [11].

However, despite being used in such consensuses and being considered a classic pathological biomarker of nephrotoxicity, creatinine often does not directly reflect tubular function [12,13]. Creatinine has important limitations, including its susceptibility to interference by several physiological and pathological factors [10,14], as well as to changes in serum or urinary values caused by changes in hydration status, without kidney damage [15]. Concerningly, serum creatinine often increases only after significant loss of renal function [16]. In 2007, kidney injury molecule-1 (KIM-1) was approved by the Food and Drug Administration and the European Medicines Agency, along with a panel of six other urinary proteins, as a potential biomarker for acute drug-induced nephrotoxicity [17,18].

The poor specificity and sensitivity of classic pathological biomarkers [15,19] can ultimately lead to misinformed conclusions and late diagnoses, causing further damage. Nephrotoxicity remains a limiting factor in cisplatin-based treatment regimens. Moreover, timely detection of kidney injury can positively impact morbidity and mortality; therefore, finding ways to improve the therapy of these patients is paramount. Thus, this study aimed to compare the tools used to assess and monitor renal function in these patients.

## 2. Results

### 2.1. Participants

**Figure 1:**
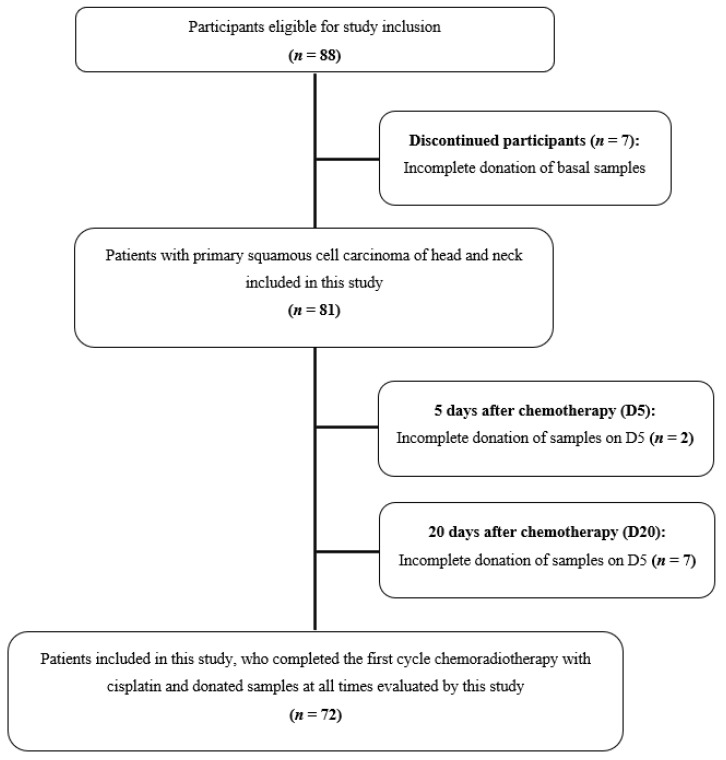
Patients with head and neck cancer from the Clinical Oncology Department (*Hospital de Clínicas*, University of Campinas, Brazil), who were included or removed from this study.

A total of 88 participants (*n* = 88) with head and neck cancer were eligible for inclusion in this study. Of these, 7 were removed as they lacked the required samples needed to carry out the study (Figure 1). Therefore, 81 participants were included in the study. Of these, only 72 performed the complete donation of samples after the first cycle of chemotherapy (two participants did not donate the D5 samples and 7 did not donate the D20 samples) (Figure 1).

Participants were mostly male (90.1%), Caucasian (76.5%), approximately 58 years old, with tumors located mainly in the oral cavity (38.3%) and at an advanced stage (stage IV, 75.3%). Most of them also declared a history of heavy smoking and alcoholism (76.5% and 47.5%, respectively), (Table 1).

### 2.2. Potentially Nephrotoxic Drugs

A total of 24 participants (29.6%) were using potentially nephrotoxic long-term medication, and some consumed more than one medication. Of these 24 participants, 12 had some degree of nephrotoxicity. The three most commonly used drugs were losartan (*n* = 3), hydrochlorothiazide (*n* = 3), and omeprazole (*n* = 3). Further details are described in Table A1 of Appendix A.

### 2.3. Chemotherapy Regimen and Toxicities

Only approximately 60% of the investigated participants (*n* = 48) maintained the same protocol and dose of cisplatin after the first cycle of chemotherapy. Moreover, four participants had no information about treatment follow-up.

Of the 29 participants who had some change in chemotherapy regimen after the first treatment cycle, 15 (51.7%) had nephrotoxicity as the reason for this change (Table 2). This change could have been due to either reduction in the dose of cisplatin, change to another platinum derivative, or treatment interruption. To verify whether other factors could also influence this outcome, the demographic and clinical profile of these participants was verified. Results showed the mean participant age was very close to the general mean (59.9 versus 58.1, respectively), and that the most frequent Karnofsky Performance Status (KPS) was 90% (data not shown).

Nephrotoxicity was considered by the medical team as the reason for changes in the treatment protocol when estimated glomerular filtration rate (eGFR) ≤60 mL/min/1.73 m^2^.

The Common Terminology Criteria for Adverse Events (CTCAE) grade of renal adverse reactions was one of the criteria used by the oncologists of this outpatient clinic for follow-up and treatment related decisions. When looking specifically for the fifteen participants who underwent a change in chemotherapy regimen after the first cycle due to nephrotoxicity, eleven participants still had some degree of increased serum creatinine and reduced creatinine clearance on D20. Though, when considering the AKI classifications RIFLE or AKIN, there were seven and six participants, respectively (Table 3).

### 2.4. Biomarkers Assessment AKI Assessment

#### 2.4.1. Serum Creatinine, Creatinine Clearance, Urea, Sodium, Potassium, Magnesium and Calcium

A significant change was observed in all nephrotoxicity traditional biomarkers evaluated in this study (serum creatinine, creatinine clearance, urea, sodium, potassium, magnesium, and calcium) (Table 4). This change was more significant after the first five days of treatment; however, after twenty days, most of these biomarkers had results close to those seen before the start of treatment. This can be expected as this was the time-point these patients were evaluated regarding their follow-up to the second treatment cycle.

#### 2.4.2. eGFR Determination

Almost 90% of participants had a decreased eGFR on D5 following the first treatment cycle compared to their baseline values, regardless of whether the estimation equation was used. Despite some slight signs of recovery, on D20, about 70% still had decreased eGFR (Table 5). The mean eGFR remained above 60 mL/min/1.73 m^2^; therefore, it was not considered a clinically significant reduction for all participants (Table 6).

The variations between the estimates obtained by the different equations were not statistically considered at either of the two time-points.

#### 2.4.3. Kidney Injury Molecule-1 (KIM-1)

The total group of participants was subdivided into those who had any degree of renal impairment (according to the AKIN or RIFLE classifications for AKI, or according to CTCAE for increased serum creatinine), and those who did not. When comparing the plasma KIM-1 values on D5 between the two groups, it is possible to verify whether there was a statistically significant difference between the two groups using the CTCAE or AKIN classification (*p* > 0.05, in both cases) (Table 7).

Participants were divided into those who consumed some other potentially nephrotoxic drug (*n* = 24) or those who did not (*n* = 49). There was a statistically significant difference between their KIM-1 values five days after treatment (*p* < 0.05, Mann–Whitney test).

Table 8 divides the same participants as shown in Table 7, this time according to their KIM-1 values. In this case, it was also possible to verify that the participants who presented the most severe grades of AKI/renal adverse events on D5 were all among the 25% (*n* = 17) of patients with a higher plasma KIM-1 values (absolute values ≥ 13.3 pg/mL). However, among these 17 participants, 6 (35.3%) were still classified as grade 0 according to CTCAE or AKIN, and 4 (23.5%) were classified as grade 0 according to the RIFLE classification.

### 2.5. Nephrotoxicity Assessment

#### 2.5.1. CTCAE

Table 9 presents the highest degree of renal adverse event severity determined during the first chemotherapy cycle, according to the CTCAE. The majority of participants who presented with such adverse events had a lower level of severity (prevalence of grade 1), except for those with a reduction in creatinine clearance (predominant grade of 2).

#### 2.5.2. RIFLE, AKIN and KDIGO

On D5, according to the RIFLE classification, 40 participants (50.6%) were diagnosed with some stage of AKI, whereas the AKIN classification determined 33 participants (41.8%). On D20, these numbers were lower: according to the RIFLE and AKIN classifications, 12 (16.7%) and 9 (12.5%) participants were diagnosed with some stage of AKI, respectively (Table 10). At the same time, 15 participants had some difference between RIFLE and AKIN classifications (meaning only one of these two criteria was able to identify the participant’s AKI stage). In the first treatment cycle, only 32 participants (58.2%) had no degree of AKI.

The AKI classification of these participants was also verified according to the KDIGO guideline; since this system represents the combination of RIFLE and AKIN criteria, the data of KDIGO classification is presented in Table A2 of Appendix A. Although the KDIGO staging system represents the combination of RIFLE and AKIN criteria, in this study, the KDIGO system identified fewer patients with AKI than either of the other two classifications (Appendix A, Table A1).

The renal laboratory markers evaluated showed statistically significant changes on the D5. This was unlike those observed in the AKI classification, in which, on D20, most participants had results close to those found in the baseline period. During this period, the most serious renal adverse reactions were increased serum creatinine, reduced creatinine clearance, and hyponatremia (Table 7).

**Figure 2:**
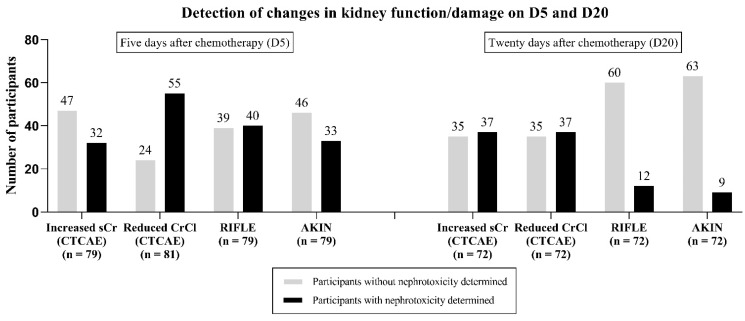
Comparison of the potential of renal biomarkers (serum creatinine and creatinine clearance according to CTCAE) and AKI classifications (RIFLE, AKIN) in identifying altered renal function/damage on D5 in patients treated with cisplatin. Participants who did not have any of the marker values in question were disregarded in this evaluation. D5, 5th day from the first cycle of chemotherapy with cisplatin; sCr, serum creatinine; CrCl, creatinine clearance.

A comparison of the potential of the evaluated biomarkers (serum creatinine and creatinine clearance according to CTCAE) and of the adopted AKI classifications (RIFLE and AKIN) was made to detect changes in kidney function or damage by cisplatin on D5 (Figure 2). As there are still no established reference values for KIM-1, this was not considered in this case.

For serum creatinine, those who had a CTCAE grade for serum creatinine increase of ≤1 were considered as altered; for creatinine clearance, estimated by Cockroft–Gault, those who had a CTCAE grade for creatinine clearance decrease of ≤1 were considered as altered; for the RIFLE classification, those who had grade ≤R were considered as altered; for the AKIN classification, those with grade ≤1 were considered as altered. For all cases, participants who had baseline values higher than those found on D5 were not considered to have renal impairment by the parameter.

## 3. Discussion

To the best of our knowledge, this is the first study to investigate the differences between the main proposals for renal evaluation and follow-up in patients with head and neck cancer treated with cisplatin. Nephrotoxicity is the most burdensome adverse effect of cisplatin, affecting 25% to 35% of patients with treatment-limiting consequences [23]. Despite extensive research in recent years to mitigate the cytotoxic effects of cisplatin on renal tissue, little progress has been made to date [24,25]. Therefore, new studies that evaluate the best method for timely detection of AKI associated with cisplatin use and preventive strategies are of great relevance.

First of all, it is interesting to note that although cisplatin is also used in the treatment of other solid neoplasms, this research had a special focus on patients with head and neck cancer due to some peculiarities of this patient profile that makes them more susceptible to AKI: (1) Patients with head and neck cancer are usually diagnosed with locally advanced disease: this often implies the need to use a nasogastric tube as support to avoid secondary nutritional deficiencies [26]. Malnutrition can either be a consequence of the tumor location itself (which sometimes makes swallowing difficult) or of radiotherapy toxicity (which can cause dysphagia, xerostomia, and mucositis) [27]. In addition, because of the difficulty in swallowing, they usually present a significant weight loss throughout the treatment, directly impacting the estimated glomerular filtration rate; (2) These patients are commonly at advanced ages at diagnosis: in addition to being itself a risk factor for AKI [28], advanced age is often associated with a higher incidence of comorbidities (such as hypertension and diabetes) that are known to also contribute to kidney damage [29]. As an aggravating factor, some participants were exposed to further drugs with the potential to impair kidney function due to these comorbidities. The consumption of two or more nephrotoxic co-medications is known to be one of the risk factors associated with cisplatin-induced AKI [8,30]. In that regard, it was found that 12 of the 24 participants who used some other potentially nephrotoxic drug had some degree of nephrotoxicity. Given this concomitant use, it is unlikely to clearly determine the individual contribution of each agent to the development of AKI; however, the serum creatinine and creatinine clearance profiles of these 12 participants before the use of cisplatin showed that only one participant already had abnormal values. Therefore, it is possible to infer that for the other 11 patients, the nephrotoxicity outcome was mainly caused by possible drug interactions between cisplatin and other drugs.

With regard to strategies to prevent nephrotoxicity, the use of hydration during chemotherapy is one of the most recommended ones [31], usually reducing the incidence of severe kidney damage. Therefore, in this study, among those who had renal adverse events after treatment, there was a prevalence of grade 1 for increased serum creatinine (mean increase of 60% compared to baseline), and of grade 2 for a reduction in creatinine clearance (mean decrease of 26% compared to baseline) according to CTCAE. This frequency of adverse reactions is similar to those verified in the study by Visacri et al., whose patients also had head and neck cancer and underwent the same treatment protocol [32]. However, even some participants who appeared to have only mild degrees of acute nephrotoxicity did not recover adequately and worsened in the following days, requiring a protocol change for the next treatment cycle. Therefore, even the mildest cases of renal adverse reactions should be followed with caution.

A state of acute nephrotoxicity was also indicated, on the fifth day after treatment, by marked and significant changes in laboratory markers. This acute behavior, considered to be relatively expected in patients treated with cisplatin [33], was only recovered in 25 participants (34.7% of the 72 participants for whom data were available on D20). The remaining 47, when compared to their respective baseline data, still had abnormal values of serum creatinine and/or creatinine clearance after 20 days of chemotherapy with cisplatin.

Another expected manifestation of tubular damage caused by cisplatin is serum electrolytic alterations [23,33]. In this study, the most frequent disturbance was hyponatremia, followed by hypocalcemia and hypomagnesemia. Although hyponatremia is rarely reported, hypomagnesemia is recognized as one of the most common clinical presentations in cisplatin-induced nephrotoxicity, with a prevalence of 40% or more [34,35].

Tubular dysfunction after cisplatin exposure may also lead to AKI. As more severe stages of AKI have already been shown to be associated with increased mortality [36,37], staging is essential for prompt management. In addition, it is equally important to choose the most appropriate eGFR equation for this population; however, since most of the equations commonly used in adults were derived in the steady state, they are less accurate in non-stationary cases, such as AKI [38]. Furthermore, a second difficulty when using the AKIN or RIFLE classifications, as pointed out by the authors of [10], is the use of urinary output as one of its parameters. Despite having a faster response in the face of changes in GFR [11], these data are hardly evident outside of inpatient regimens.

Since the AKIN consensus was developed from the RIFLE classification with the aim of identifying previously undiagnosed patients with minor changes in serum creatinine, it could be expected that more participants would be detected according to this new criterion. However, in this study, more participants were diagnosed with AKI according to the RIFLE consensus (Table 7). This likely occurred because in addition to considering serum creatinine and urinary output, RIFLE also considers the decrease in GFR [9] (the latter parameter is not considered by the AKIN) [10]. While some participants showed an increase in serum creatinine, this was not significant according to the criteria established by AKIN. However, the large weight loss experienced by these patients is also reflected in the eGFR; therefore, this seems to be a more adequate care and management option for these patients.

The lack of consensus on which of these two staging definitions should be adopted compromises the estimates of the incidence and prevalence of AKI, as well as their comparison between different studies. Despite this divergence between classifications, all fifteen patients who underwent changes in the chemotherapy regimen after the first cycle (due to nephrotoxicity) were classified with some degree of AKI/renal adverse reaction severity by one of the three classifications considered. Therefore, for the best management of these patients, the Kidney Disease: Improving Global Outcomes (KDIGO) clinical practice guidelines recommend that for AKI diagnosis, the RIFLE and AKIN criteria should be used concomitantly, classifying the patient according to the criterion that gives a more serious stage [11].

Even if this strategy is adopted, it should be noted that both the AKIN and RIFLE consensuses have intrinsic limitations. Firstly, being mainly based on serum creatinine may lead to mistaken conclusions or late detection of kidney damage. A proposal for better stratification of patients with AKI, as pointed out by the report of the 10th Acute Dialysis Quality Initiative conference, is the combined use of markers that assess renal function (such as serum creatinine), and those that reflect renal damage (such as KIM-1) [39]. This would allow the diagnosis even when one remains unaltered, allowing a more accurate determination of the AKI etiology [39]. As cisplatin is known to cause tubular damage, and considering the specificity of KIM-1 for this type of lesion [40], it was decided to evaluate the KIM-1 expression in these participants. Some patients already had slightly high concentrations even before starting treatment. In addition, the mean baseline KIM-1 was about eight times higher than that found in a study of healthy volunteers (465.3 in this study versus 64.4 in healthy volunteers [41]). This may be a reflection of unidentified subclinical kidney injury or asymptomatic chronic kidney disease; moreover, the participants in the present study had other comorbidities that are known to also affect kidney function. These included hypertension (23.5% of total participants), and diabetes (11.1% of total participants), in addition to being of advanced age.

Clearly, it is still too premature to draw any conclusions; though, when comparing KIM-1 expression between participants who did not have any degree of nephrotoxicity and those who had, according to the CTCAE or AKIN, KIM-1 was identified as a potential biomarker of kidney damage in patients treated with cisplatin (*p* < 0.05, in both cases on D5). However, when the classification adopted was RIFLE, the KIM-1 values were not significant enough to distinguish between those with AKI and those without AKI (*p* = 0.0780). The same was observed in a study by Pavkovic et al. [40], who evaluated the expression of KIM-1 in a longitudinal cohort of 108 patients with malignant mesothelioma receiving cisplatin intraoperative therapy. They showed that the biomarker was altered after treatment but was not able to differentiate between the two groups of patients [40].

An interesting observation was made in the present work when separating participants who consumed other potentially nephrotoxic drugs from those who did not. In this case, there was a statistically significant difference in plasma KIM-1 expression before and after treatment in the group that did not report consuming other potentially nephrotoxic drugs (*p* < 0.05) (Appendix A, Table A1).

It is worth noting that some of the other studies that showed favorable results for KIM-1 opted to use urine samples [42,43], mainly owing to the minimally invasive sample collection and their good correspondence with renal alterations. Conversely, in this study, plasma samples were used. The main reason for this is that urinary biomarker normalization by urinary creatinine excretion remains controversial, as suggested by most current studies [44,45]. Changes in the rate of creatinine excretion caused by changes in glomerular filtration or tubular secretion could result in misleading conclusions regarding the marker [44,45]. In these cases, the method considered the gold standard to measure the current GFR is based on determining the clearance of the exogenous filtration marker inulin [38]. In addition to greater precision, the advantage of using this marker as a reference in clinical research is the possibility of identifying patients with suspected AKI, even when the serum creatinine concentration remains within the normal range [16,46]. However, in clinical practice, the use of these exogenous markers is expensive, impractical, invasive, and time-consuming [47]. For this reason, we chose to use plasma-derived KIM-1 samples. These can be obtained even in anuric patients and contain a reduced number of interfering proteins [48]. Nevertheless, studies comparing KIM-1 detection and expression profiles in these two types of samples are needed.

Furthermore, the standard deviation found in the KIM-1 test was high in all groups. Regardless of the reason, it should be noted that this high variability has a negative impact on the sensitivity and specificity of the KIM-1 test [48].

Although we presented, for the first time, the differences between the main proposals for renal evaluation in patients with head and neck cancer treated with cisplatin, our work has some limitations. Since it was not possible to assess inulin clearance (considered the gold standard), it was not possible to determine how close or far from this marker the KIM-1 performance was in patients treated with cisplatin.

### Future Perspectives

Even many years after its discovery as a drug, little is known about the long-term consequences of cisplatin treatment [8]. Among other reasons, this occurs mainly due to a large number of participants not proceeding to the second course of cisplatin [49]. This was also observed in this study, mainly due to renal impairment. Furthermore, considering the specific case of patients with head and neck cancer, as many are diagnosed in the advanced stages of the disease, the survival rate is usually not high [50], making follow-up difficult in most cases.

Equally relevant is the question regarding the time of KIM-1 samples collection. A deeper understanding of the pathophysiological role of proteins is needed to establish the correct timing for quantification, aiming to determine whether it would work better as a predictor biomarker or as a diagnostic biomarker. Therefore, although KIM-1 may have surpassed the sensitivity of traditional biomarkers in previous studies [40,51], there is still a long way to go before it can be clinically implemented as a specific indicator of cisplatin-induced AKI.

Finally, despite its important limitations, creatinine continues to be frequently used to estimate GFR owing to the wide availability of assays for its detection, which are generally easy to conduct and at a low cost. Therefore, to enable its use in clinical practice, improving and making the detection techniques of KIM-1 cheaper is as important as establishing its reference values which are not yet defined. The best way to monitor the renal function of these participants before, during, and after cisplatin therapy is still unknown, but it is hoped that the present study will contribute to building this knowledge.

## 4. Materials and Methods

### 4.1. Study Design and Ethical Considerations

This was a retrospective, cohort study. This study was reported in accordance with the STROBE Statement and Checklist for Cohort Studies available from the STROBE website (https://www.strobe-statement.org/, accessed on 26 September 2022). The study was conducted in accordance with the Declaration of Helsinki and was approved by the Ethics Committee of the University of Campinas (protocol code: 65397517.7.0000.5404, 8 February 2021).

### 4.2. Setting and Participants

This study was conducted in the Clinical Oncology Department of the Hospital de Clínicas, University of Campinas (HC-UNICAMP), a large tertiary teaching hospital in Campinas, Brazil.

Patients were selected by consecutive non-probabilistic sampling during the period from May 2017 to December 2018. Patients were included in the study if they were between 18 and 80 years old, diagnosed with primary SCC of the head and neck, had creatinine clearance ≥ 60 mL/min/1.73 m^2^, performed the first cycle of cisplatin chemotherapy concomitantly with conventional radiotherapy, and agreed to participate. Participants who did not provide the necessary sample for the study were excluded.

Although this study only considered the first cycle of treatment, the complete protocol consisted of three cycles of chemotherapy with cisplatin (doses ranged from 80 to 100 mg/m^2^) every 21 days. Concurrently, patients received a total dose of 70 Gy of radiation therapy divided into 35 daily applications of 2 Gy administered 5 days/week for 7 weeks. On the day of chemotherapy, as part of the protocol for the prevention of adverse reactions, the patients also received vigorous hydration, diuresis, and prophylaxis for acute emesis. For treatment and prevention of delayed emesis, patients received 10 mg of metoclopramide every 6 h and 8 mg dexamethasone every 12 h for three consecutive days after the chemotherapy sessions.

### 4.3. Data Collection

Demographics (age at diagnosis, sex, and ethnicity) and clinical data (smoking [20,21], alcoholism history [22], tumor location, stage, and evaluation according to the KPS [52]) were collected. Long-term medications (used for more than one month) were also investigated to assess whether the AKI of these participants was also influenced by consumption of other potentially nephrotoxic agents [30,53].

All information was collected from the patients’ own report records during individual face-to-face interviews or through clinical history in electronic medical records.

### 4.4. Kidney Assessment

Cisplatin-induced nephrotoxicity was assessed using serum creatinine, estimated creatinine clearance (determined by the Cockroft–Gault equation), urea, sodium, magnesium, calcium, and potassium. Baseline values were compared to values at 5 and 20 days after the first cycle of cisplatin administration (D5 and D20, respectively), and the severity of toxicities was classified according to the CTCAE (version 4.03) [54]. AKI was classified according to RIFLE [9], AKIN [10] and KDIGO [11] criteria. The eGFR was calculated and compared using the Cockroft–Gault [55], Modification of Diet in Renal Disease [56], and Chronic Kidney Disease Epidemiology Collaboration [57] equations. The Cockroft–Gault equation was adopted for RIFLE and AKIN classifications.

KIM-1 was detected in plasma samples collected before treatment and on D5, using the Human TIM-1 ELISA Kit (ThermoFisher, Waltham, MA, USA), following the manufacturer’s instructions.

### 4.5. Statistical Analysis

For the analysis of clinical, demographic, and adverse reaction data, absolute frequencies/percentages, and measures of position (mean) and dispersion (standard deviation) are presented. Nephrotoxicity markers were compared at baseline, D5, and D20 using Friedman’s test. The Wilcoxon test was used to compare KIM-1 levels at baseline and D5. Data normality was tested using the Shapiro–Wilk test. The significance level adopted for all analyses was set at 5% (*p* < 0.05). All statistical analyses were performed using GraphPad Prism v.9.1.0 software. for Windows (GraphPad Software, Inc., San Diego, CA, USA).

## 5. Conclusions

The high incidence of patients with cisplatin nephrotoxicity reinforces the need for a more complete renal evaluation of these patients before, during, and after therapy. More participants were diagnosed with AKI according to the RIFLE consensus. KIM-1 was identified as a possible biomarker of kidney damage in patients treated with cisplatin when comparing its expression between participants who did not have any degree of nephrotoxicity and those who had, according to the CTCAE or AKIN, but not when RIFLE was adopted. It is still necessary to understand the profiles of different biomarkers together instead of just one or two isolated candidates.

## Figures and Tables

**Table 1:** Demographic and clinical data of included participants (*n* = 81).

Variable	Participants (*n* = 81)
**Age at diagnosis (mean ± SD, years)**	58.12 ± 7.62
**Gender (*n*, %)**	
Male	73 (90.1)
Female	8 (9.9)
**Ethnicity (*n*, %)**	
Caucasian	62 (76.5)
Non-Caucasian	19 (23.5)
**Smoking category [20,21] (*n*, %)**	
Never smoked	10 (12.3)
Light smoker	4 (4.9)
Moderate smoker	5 (6.2)
Heavy smoker	62 (76.5)
**Drinking category [22] (*n*, %)**	
Abstainer	11 (13.6)
Light drinker	7 (8.6)
Moderate drinker	5 (6.2)
Heavy drinker	21 (25.9)
Very heavy drinker	37 (45.7)
**Never smoked and abstainer (*n*, %)**	6 (7.4%)
**Tumor site (*n*, %)**	
Oral cavity	31 (38.3)
Hypopharynx	9 (11.1)
Hypopharynx and larynx	1 (1.2)
Larynx	18 (22.2)
Oropharynx	19 (23.5)
Not assessed	3 (3.7%)
**Tumor stage (*n*, %)**	
I	0 (0.0)
II	7 (8.6)
III	12 (14.8)
IV	61 (75.3)
Not assessed	1 (1.2)
**KPS (*n*, %)**	
100	12 (14.8)
90	51 (63.0)
80	12 (14.8)
70	5 (6.2)
60	1 (1.2)
**Comorbidities**	
Hypertension	19 (23.5)
Diabetes	9 (11.1)

*n*, absolute number of patients; KPS, Karnofsky Performance Status; SD, standard deviation.

**Table 2:** Reasons for changing treatment after the first cycle of treatment with cisplatin (*n* = 29).

Reasons for Changing Treatment *	(*n*, %)
Nephrotoxicity	15 (51.7)
Myelotoxicity	1 (3.4)
Gastrointestinal toxicities	5 (17.2)
KPS	1 (3.4)
Other	10 (34.5)

*n*, absolute number of patients; KPS, Karnofsky Performance Status. * Some participants had more than one reason for changing their treatment approach.

**Table 3:** Profile of participants who underwent changes in chemotherapy regimen after the first cycle due to nephrotoxicity (*n* = 15).

Fifth Day after Chemotherapy (D5) (*n*, %)								Twentieth Day after Chemotherapy (D20) (*n*, %)							
CTCAE—Increased Serum Creatinine (*n* = 15)		CTCAE—Reduced Creatinine Clearance (*n* = 15)		RIFLE (*n* = 15)		AKI (*n* = 15)		CTCAE—Increased Serum Creatinine (*n* = 13 *)		CTCAE—Reduced Creatinine Clearance (*n* = 13 *)		RIFLE (*n* = 13 *)		AKIN (*n* = 13 *)	
**Grade 1**	7 (46.7)	**Grade 1**	1 (6.7)	**R**	7 (46.7)	**1**	11 (73.3)	**Grade 1**	3 (23.3)	**Grade 1**	3 (23.3)	**R**	4 (30.8)	**1**	7 (53.8)
**Grade 2**	5 (33.3)	**Grade 2**	10 (66.7)	**I**	5 (33.3)	**2**	0 (0.0)	**Grade 2**	8 (61.5)	**Grade 2**	8 (61.5)	**I**	2 (15.4)	**2**	0 (0.0)
**Grade 3**	2 (13.3)	**Grade 3**	1 (6.7)	**F**	2 (13.3)	**3**	2 (13.3)	**Grade 3**	0 (0.0)	**Grade 3**	0 (0.0)	**F**	0 (0.0)	**3**	0 (0.0)
**Grade 4**	0 (0.0)	**Grade 4**	2 (13.3)	**L**	0 (0.0)			**Grade 4**	0 (0.0)	**Grade 4**	0 (0.0)	**L**	0 (0.0)		
Changes not considered relevant **: 1 (6.7)		Changes not considered relevant **: 1 (6.7)		**E**	0 (0.0)	Changes not considered relevant **: 2 (13.3)		Changes not considered relevant **: 2 (15.4)		Changes not considered relevant **: 2 (15.4)		**E**	0 (0.0)	Changes not considered relevant **: 6 (46.2)	
				Changes not considered relevant **: 1 (6.7)								Changes not considered relevant **: 7 (53.8)			

AKI, acute kidney injury; AKIN, Acute Kidney Injury Network; E, end-stage kidney disease; F, failure; I, injury; *n*, absolute number of patients; R, risk; RIFLE, Risk, Injury, Failure, Loss, and End-stage kidney disease. * In D20, *n* = 13 because 2 of these 15 participants did not collect samples on D20. ** Participants who may had some change, but not enough to be classified by this criterion.

**Table 4:** Renal laboratory markers before, five and twenty days after the first cycle of chemotherapy with cisplatin.

Renal Laboratory Markers (Mean ± SD)	Baseline	D5	D20	*p*-Value *
Serum creatinine (mg/dL) (*n* = 70)	0.8 ± 0.2	1.3 ± 0.9	0.9 ± 0.3	<0.0001
Creatinine clearance ** (mL/min) (*n* = 70)	87.4 ± 25.5	63.3 ± 23.2	77.8 ± 26.5	<0.0001
Urea (mg/dL) (*n* = 68)	29.9 ± 11.9	53.1 ± 21.7	34.0 ± 10.4	<0.0001
Sodium (mEq/L) (*n* = 59)	136.6 ± 3.2	131.9 ± 3.6	135.0 ± 4.3	<0.0001
Potassium (mEq/L) (*n* = 59)	4.5 ± 0.5	4.3 ± 0.7	4.6 ± 0.5	<0.001
Magnesium (mEq/L) (*n* = 44)	1.7 ± 0.2	1.7 ± 0.2	1.5 ± 0.3	<0.0001
Calcium (mg/dL) (*n* = 58)	9.7 ± 0.8	9.3 ± 0.8	9.1 ± 0.7	<0.0001

D5, 5th day after the first cycle of cisplatin chemotherapy; D20, 20th day after the first cycle of cisplatin chemotherapy; *n*, absolute number of patients. * Friedman test. ** Estimated by Cockroft–Gault equation.

**Table 5:** Changes in eGFR calculated according to three different equations (Cockroft–Gault, Modification of Diet in Renal Disease, and Chronic Kidney Disease Epidemiology Collaboration).

Changes in eGFR	D5 (*n* = 79)			D20 (*n* = 72)		
	eGFR-CG (*n*, %)	eGFR-MDRD (*n*, %)	eGFR-CKD-EPI (*n*, %)	eGFR-CG (*n*, %)	eGFR-MDRD (*n*, %)	eGFR-CKD-EPI (*n*, %)
Mean ± SD (mL/min/1.73 m^2^)	63.27 ± 25.0	79.6 ± 33.6	75.9 ± 29.2	77.6 ± 34.0	101.0 ± 46.0	92.1 ± 35.4
Reduced eGFR *	71 (89.9)	70 (88.6)	70 (88.6)	55 (76.4)	50 (69.4)	49 (68.0)
Unchanged eGFR *	1 (1.2)	0	0	0	1 (1.4)	1 (1.4)
Increased eGFR *	7 (8.9)	9 (11.4)	9 (11.4)	17 (23.6)	21 (29.2)	22 (30.5)
*p*-value **	>0.9999			0.8889		

CKD-EPI, Chronic Kidney Disease Epidemiology Collaboration; CG, Cockroft–Gault; D5, 5th day after the first cycle of cisplatin chemotherapy; D20, twentieth day after the first cycle of cisplatin chemotherapy; eGFR, estimated glomerular filtration rate; MDRD, Modification of Diet in Renal Disease; *n*, absolute number of patients. * Comparison with respective baseline values. ** Friedman test.

**Table 6:** eGFR calculated according to three different equations (Cockroft–Gault, Modification of Diet in Renal Disease, and Chronic Kidney Disease Epidemiology Collaboration) at at baseline, D5 and D20.

eGFR (Mean ± SD) (mL/min/1.73 m^2^)	Baseline (*n* = 81)	D5 (*n* = 79)	D20 (*n* = 72)
eGFR-CG	83.37 ± 25.5	63.27 ± 25.0	77.6 ± 34.0
eGFR-MDRD	112.7 ± 30.4	79.6 ± 33.6	101.0 ± 46.0
eGFR-CKD-EPI	99.0 ± 17.9	75.9 ± 29.2	92.1 ± 35.4

CKD-EPI, Chronic Kidney Disease Epidemiology Collaboration; CG, Cockroft–Gault; D5, 5th day after the first cycle of cisplatin chemotherapy; D20, twentieth day after the first cycle of cisplatin chemotherapy; eGFR, estimated glomerular filtration rate; MDRD, Modification of Diet in Renal Disease; *n*, absolute number of patients; SD, standard deviation.

**Table 7:** Plasma KIM-1 before and five days after the first cycle of treatment with cisplatin (mean ± standard deviation), according to the division between who had any degree of renal impairment and those who did not.

Plasma KIM-1 (pg/mL)					
CTCAE-Increased Serum Creatinine					
**Baseline**			**D5**		
**Grade 0** **(*n* = 38)**	**Grade ≥1** **(*n* = 29)**	*p*-value *	**Grade 0** **(*n* = 38)**	**Grade ≥ 1** **(*n* = 29)**	*p*-value *
507.5 ± 1335.0	426.1 ± 934.6	0.1583	361.8 ± 777.1	598.6 ± 704.6	<0.05
**RIFLE**					
**Baseline**			**D5**		
**Grade 0** **(*n* = 57** **)**	**Grade** **R, I, F, L or E** **(*n* = 34)**	*p*-value *	**Grade 0** **(*n* = 57** **)**	**Grade** **R, I, F, L or E** **(*n* = 34)**	*p*-value *
508.8 ± 1272.9	619.3 ± 1451.5	0.3276	401.0 ± 719.2	681.0 ± 987.3	0.0780
**AKIN**					
**Baseline**			**D5**		
**Grade 0** **(*n* = 38)**	**Grade ≥ 1** **(*n* = 3)**	*p*-value *	**Grade 0** **(*n* = 38)**	**Grade ≥ 1** **(*n* = 3)**	*p*-value *
509.5 ± 1354.3	453.3 ± 688.3	0.6847	366.6 ± 755.6	1679.0 ± 933.2	<0.05

AKIN, Acute Kidney Injury Network; CTCAE, Common Terminology Criteria for Adverse Events (assessment according to increase in serum creatinine); D5, 5th day after the first cycle of cisplatin chemotherapy; KIM-1, Kidney Injury Molecule-1; *n*, absolute number of patients; RIFLE, Risk, Injury, Failure, Loss, and End-stage kidney disease; * Mann–Whitney U test.

**Table 8:** Frequency of CTCAE (for increased serum creatinine, RIFLE and AKIN grades according to their plasma KIM-1 values five days after chemotherapy) (*n* = 68).

	Plasma KIM-1 Fifth Day after Chemotherapy (D5)			
	0–119.0 pg/mL (*n* = 17, 25%)	119.1–204.4 pg/mL (*n* = 17, 25%)	204.5–413.2 pg/mL (*n* = 17, 25%)	≥413.3 pg/mL (*n* = 17, 25%)
**CTCAE-Increased serum creatinine (D5) (*n*, %)**				
Grade 0	11 (64,7)	12 (70.6)	9 (52.9)	6 (35.3)
Grade 1	4 (23.5)	3 (17.6)	5 (29.4)	7 (41.2)
Grade 2	1 (5.9)	2 (11.8)	3 (17.6)	1 (5.9)
Grade 3	0 (0.0)	0 (0.0)	0 (0.0)	3 (17.6)
Not assessed	1 (5.9)	0 (0.0)	0 (0.0)	0 (0.0)
**RIFLE (D5) (*n*, %)**				
AKI not determined by this criterion	9 (52.9)	11 (64,7)	9 (52.9)	4 (23.5)
Risk (R)	6 (35.3)	4 (23.5)	5 (29.4)	9 (52.9)
Injury (I)	1 (5.9)	2 (11.8)	3 (17.6)	1 (5.9)
Failure (F)	0 (0.0)	0 (0.0)	0 (0.0)	3 (17.6)
Not assessed	1 (5.9)	0 (0.0)	0 (0.0)	0 (0.0)
**AKIN (D5) (*n*, %)**				
AKI not determined by this criterion	10 (58.8)	12 (70.6)	10 (58.8)	6 (35.3)
Grade 1	6 (35.3)	5 (29.4)	7 (41.2)	8 (47.1)
Grade 3	0 (0.0)	0 (0.0)	0 (0.0)	3 (17.6)
Not assessed	1 (5.9)	0 (0.0)	0 (0.0)	0 (0.0)

AKI, acute kidney injury; AKIN, Acute Kidney Injury Network; CTCAE, Common Terminology Criteria for Adverse Events (assessment according to increase in serum creatinine); D5, 5th day after the first cycle of cisplatin chemotherapy; KIM-1, Kidney Injury Molecule-1; *n*, absolute number of patients; RIFLE, Risk, Injury, Failure, Loss, and End-stage kidney disease.

**Table 9:** Most severe degree of renal adverse events presented during the first cycle of chemotherapy with cisplatin according to CTCAE (version 4.03).

Renal Adverse Events (*n*, %)	Severity-CTCAE			
	Grade 1	Grade 2	Grade 3	Grade 4
Increased serum creatinine (*n* = 79)	21 (26.6)	8 (10.1)	3 (3.8)	0 (0.0)
Reduced creatinine clearance (*n* = 81)	28 (34.6)	31 (38.3)	2 (2.5)	2 (2.5)
Hyponatremia (*n* = 81)	42 (51.9)	-	16 (19.8)	2 (2.5)
Hypokalemia (*n* = 81)	4 (4.9)	0 (0.0)	0 (0.0)	0 (0.0)
Hypomagnesemia (*n* = 76)	15 (19.7)	2 (2.6)	0 (0.0)	0 (0.0)
Hypocalcemia (*n* = 80)	16 (20.0)	3 (3.8)	0 (0.0)	0 (0.0)

*n*, absolute number of patients.

**Table 10:** AKI stage on the fifth and twentieth days after the first cycle of chemotherapy with cisplatin, according to the AKIN and RIFLE classifications.

Fifth Day after Chemotherapy (D5) (*n*, %)						Twentieth Day after Chemotherapy (D20) (*n*, %)					
RIFLE (*n* = 79)		AKIN (*n* = 79)		Comparison between RIFLE and AKIN (*n* = 41)		RIFLE (*n* = 72)		AKIN (*n* = 72)		Comparison between RIFLE and AKIN (*n* = 15)	
**R**	29 (36.7)	**1**	30 (38.0)	Participants diagnosed with AKI by both classifications	32 (78.1)	**R**	10 (13.9)	**1**	9 (12.5)	Participants diagnosed with AKI by both classifications	6 (40.0)
**I**	8 (10.1)	**2**	0 (0.0)			**I**	2 (2.8)	**2**	0 (0.0)		
**F**	3 (3.8)	**3**	3 (3.8)	Participants diagnosed with AKI only by RIFLE	8 (19.5)	**F**	0 (0.0)	**3**	0 (0.0)	Participants diagnosed with AKI only by RIFLE	6 (40.0)
**L**	0 (0.0)	**NA**	46 (58.2)			**L**	0 (0.0)	**NA**	63 (87.5)		
**E**	0 (0.0)			Participants diagnosed with AKI only by AKIN	1 (2.4)	**E**	0 (0.0)			Participants diagnosed with AKI only by AKIN	3 (20.0)
**NA**	39 (49.4)					**NA**	60 (83.4)				

AKI, acute kidney injury; AKIN, Acute Kidney Injury Network; E, end-stage kidney disease; F, failure; I, injury; *n*, absolute number of patients; NA, AKI was not determined by this criterion; R, risk; RIFLE, Risk, Injury, Failure, Loss, and End-stage kidney disease. The equation used to estimate GFR in the AKI classifications adopted in this study was GC. This decision took into account that for the application of MDRD, it is necessary to have information about the patient’s race and, in the Brazilian context, this can be a limiting factor. Furthermore, as the Oncology Department (to which the participants of this study were associated) used the GC equation for therapeutic follow-up and decision-making, the same equation was adopted for the AKI classification. Note: The total number of participants who had some disagreement between the RIFLE and AKIN classifications in both periods (i.e., in D5 and D20) is fifteen participants because not all nine participants who had divergence in the D5 classifications are the same nine who had divergence in D20 (some participants are the same and others are not, totaling fifteen in both times).

## Data Availability

The datasets generated and/or analyzed during the current study are available from the Research Data Repository of the University of Campinas, https://redu.unicamp.br/dataset.xhtml?persistentId=doi:10.25824/redu/XT1BEY.

## Appendix A

**Table A1:** Continuous use medications consumed (absolute frequency and relative frequency) and profile of these participants (*n* = 24).

Continuous Use Medications(*n* = 24)	*n* (%)	Participants Profile	
		Variables	*n* (%)
**Cardiovascular agents/Diuretics**		**Age at diagnosis (mean ± SD, years**)	60.9 ± 6.3
Captopril	2 (8.0)	**Gender (*n*, %)**	
Enalapril	2 (8.0)	Male	16 (66.7)
Hydrochlorothiazide	4 (16.0)	Female	8 (33.3)
Losartan or valsartan	7 (28.0)	**Ethnicity (*n*, %)**	
Simvastatin	2 (8.0)	Caucasian	19 (79.2)
**Analgesic**		Non-Caucasian	5 (20.8)
Ibuprofen	1 (4.0)	**CTCAE (*n* = 23) (*n*, %)**	
**Benzodiazepines**		Grade 0	12 (52.2)
Clonazepam	2 (8.0)	Grade ≥ 1	11 (47.8)
Diazepam	2 (8.0)	**RIFLE-D5 (*n* = 23) (*n*, %)**	
**Proton pump inhibitor**		Grade 0	10 (43.5)
Omeprazole	2 (8.0)	Grade R, I, F, L, or E	13 (56.5)
**Others**		**AKIN-D5 (*n* = 23) (*n*, %)**	
Phenytoin	2 (8.0)	Grade 0	12 (52.2)
Ranitidine	1 (4.0)	Grade ≥ 1	11 (47.8)

AKIN, Acute Kidney Injury Network; CTCAE, Common Terminology Criteria for Adverse Events; D5, 5th day from the first cycle of chemotherapy with cisplatin; *n*, absolute number of patients. RIFLE, Risk, Injury, Failure, Loss, and End-stage kidney disease; SD, standard deviation. Note: For CTCAE, the criterion adopted was the degree of increase in the serum creatinine level.

**Table A2:** AKI stage on the fifth and twentieth days after the first cycle of chemotherapy with cisplatin, according to the KDIGO guideline.

	KDIGO	
	Fifth Day after Chemotherapy (D5) (*n* = 79) (*n*, %)	Twentieth Day after Chemotherapy (D20) (*n* = 72) (*n*, %)
**Grade 1**	27 (34.2)	9 (12.5)
**Grade 2**	-	-
**Grade 3**	7 (8.9)	-
**AKI was not determined by this criterion**	45 (57.0)	63 (87.5)

AKI, acute kidney injury; KDIGO, Kidney Disease: Improving Global Outcomes; *n*, absolute number of patients.

## References

[1] Sung H., Ferlay J., Siegel R.L., Laversanne M., Soerjomataram I., Jemal A., Bray F. (2021). Global Cancer Statistics 2020: GLOBOCAN Estimates of Incidence and Mortality Worldwide for 36 Cancers in 185 Countries. CA Cancer J. Clin..

[2] Winquist E., Agbassi C., Meyers B.M., Yoo J., Chan K.K.W. (2017). Systemic therapy in the curative treatment of head and neck squamous cell cancer: A systematic review. J. Otolaryngol.-Head Neck Surg..

[3] Bourhis J., Amand C., Pignon J.-P. (2004). Update of MACH-NC (Meta-Analysis of Chemotherapy in Head & Neck Cancer) database focused on concomitant chemoradiotherapy. J. Clin. Oncol..

[4] Li S., He X., Ruan L., Ye T., Wen Y., Song Z., Hu S., Chen Y., Peng B., Li S. (2021). Protective Effect of Mannitol on Cisplatin-Induced Nephrotoxicity: A Systematic Review and Meta-Analysis. Front. Oncol..

[5] McSweeney K.R., Gadanec L.K., Qaradakhi T., Ali B.A., Zulli A., Apostolopoulos V. (2021). Mechanisms of cisplatin-induced acute kidney injury: Pathological mechanisms, pharmacological interventions, and genetic mitigations. Cancers.

[6] Herrera-Pérez Z., Gretz N., Dweep H. (2016). A Comprehensive Review on the Genetic Regulation of Cisplatin-induced Nephrotoxicity. Curr. Genom..

[7] Miller R.P., Tadagavadi R.K., Ramesh G., Reeves W.B. (2010). Mechanisms of cisplatin nephrotoxicity. Toxins.

[8] Van Der Vorst M.J.D.L., Neefjes E.C.W., Toffoli E.C., Oosterling-Jansen J.E.W., Vergeer M.R., Leemans C.R., Kooistra M.P., Voortman J., Verheul H.M.W. (2019). Incidence and risk factors for acute kidney injury in head and neck cancer patients treated with concurrent chemoradiation with high-dose cisplatin. BMC Cancer.

[9] Bellomo R., Ronco C., Kellum J.A., Mehta R.L., Palevsky P. (2004). Acute renal failure—definition, outcome measures, animal models, fluid therapy and information technology needs: The Second International Consensus Conference of the Acute Dialysis Quality Initiative (ADQI) Group. Crit. Care.

[10] Mehta R.L., Kellum J.A., Shah S.V., Molitoris B.A., Ronco C., Warnock D.G., Levin A., Bagga A., Bakkaloglu A., Bonventre J.V. (2007). Acute kidney injury network: Report of an initiative to improve outcomes in acute kidney injury. Crit. Care.

[11] Kellum J.A., Lameire N., Aspelin P., Barsoum R.S., Burdmann E.A., Goldstein S.L., Herzog C.A., Joannidis M., Kribben A., Levey A.S. (2012). Kidney Disease: Improving Global Outcomes (KDIGO) Acute Kidney Injury Work Group. KDIGO Clinical Practice Guideline for Acute Kidney Injury. Kidney Int. Suppl..

[12] Bonventre J.V., Vaidya V.S., Schmouder R., Feig P., Dieterle F. (2010). Next-generation biomarkers for detecting kidney toxicity. Nat. Biotechnol..

[13] Makris K., Spanou L. (2016). Acute Kidney Injury: Definition, Pathophysiology and Clinical Phenotypes. Clin. Biochem. Rev..

[14] Griffin B.R., Faubel S., Edelstein C.L. (2019). Biomarkers of drug-induced kidney toxicity. Ther. Drug Monit..

[15] Waikar S.S., Betensky R.A., Emerson S.C., Bonventre J.V. (2012). Imperfect gold standards for kidney injury biomarker evaluation. J. Am. Soc. Nephrol..

[16] Dusse L.M.S., Rios D.R.A., Sousa L.P.N., Moraes R.M.M.e.S., Domingueti C.P., Gomes K.B. (2017). Biomarkers of renal function: What is currently available?. Rev. Bras. Análises Clínicas.

[17] Blank M., De Felice A., Goodsaid F., Harlow P., Hausner E., Jacobson-Kram D., Taylor W., Thompson A., Throckmorton D., Xiao S. (2009). Review of Qualification Data for Biomarkers of Nephrotoxicity Submitted by the Predictive Safety Testing Consortium Table of Contents.

[18] European Medicines Agency (2009). EMA Final Conclusion on the Pilot Joint EMEA/FDA VXDS Experience on Qualification of Nephrotoxicity Biomarkers.

[19] Van Meer L., Moerland M., Cohen A.F., Burggraaf J. (2014). Urinary kidney biomarkers for early detection of nephrotoxicity in clinical drug development. Br. J. Clin. Pharmacol..

[20] Jindal S.K., Malik S.K., Dhand R., Gujral J.S., Datta B.N. (1982). Bronchogenic carcinoma in Northern India. Thorax.

[21] Singh N., Aggarwal A.N., Gupta D., Behera D., Jindal S.K. (2012). Quantified smoking status and non-small cell lung cancer stage at presentation: Analysis of a North Indian cohort and a systematic review of literature. J. Thorac. Dis..

[22] Whitcomb D.C., Yadav D., Adam S., Hawes R.H., Brand R.E., Anderson M.A., Money M.E., Banks P.A., Bishop M.D., Baillie J. (2008). Multicenter approach to recurrent acute and chronic pancreatitis in the United States: The North American Pancreatitis Study 2 (NAPS2). Pancreatology.

[23] Peres L.A. (2013). lbert. B.; da Cunha, A.D. anta. Acute nephrotoxicity of cisplatin: Molecular mechanisms. J. Bras. Nefrol..

[24] Pabla N., Dong Z. (2008). Cisplatin nephrotoxicity: Mechanisms and renoprotective strategies. Kidney Int..

[25] Ozkok A., Edelstein C.L. (2014). Pathophysiology of cisplatin-induced acute kidney injury. Biomed Res. Int..

[26] Bossola M. (2015). Nutritional interventions in head and neck cancer patients undergoing chemoradiotherapy: A narrative review. Nutrients.

[27] Rebouças L.M., Callegaro E., Gil G.O.B., Silva M.L.G., Maia M.A.C., Salvajoli J.V. (2011). Impacto da nutrição enteral na toxicidade aguda e na continuidade do tratamento dos pacientes com tumores de cabeça e pescoço submetidos a radioterapia com intensidade modulada. Radiol. Bras..

[28] Thomas M.E., Blaine C., Dawnay A., Devonald M.A.J., Ftouh S., Laing C., Latchem S., Lewington A., Milford D.V., Ostermann M. (2015). The definition of acute kidney injury and its use in practice. Kidney Int..

[29] Prasaja Y., Sutandyo N., Andrajati R. (2014). Incidence of cisplatin-induced nephrotoxicity and associated factors among cancer patients in Indonesia. Asian Pac. J. Cancer Prev..

[30] Naughton C.A. (2008). Drug-induced nephrotoxicity. Am. Fam. Physician.

[31] Crona D.J., Faso A., Nishijima T.F., McGraw K.A., Galsky M.D., Milowsky M.I. (2017). A Systematic Review of Strategies to Prevent Cisplatin-Induced Nephrotoxicity. Oncologist.

[32] Visacri M.B., Pincinato E.D.C., Ferrari G.B., Quintanilha J.C.F., Mazzola P.G., Lima C.S.P., Moriel P. (2017). Adverse drug reactions and kinetics of cisplatin excretion in urine of patients undergoing cisplatin chemotherapy and radiotherapy for head and neck cancer: A prospective study. DARU J. Pharm. Sci..

[33] Arunkumar P.A., Viswanatha G.L., Radheshyam N., Mukund H., Belliyappa M.S. (2012). Science behind cisplatin-induced nephrotoxicity in humans: A clinical study. Asian Pac. J. Trop. Biomed..

[34] Manohar S., Leung N. (2018). Cisplatin nephrotoxicity: A review of the literature. J. Nephrol..

[35] Hodgkinson E., Neville-Webbe H.L., Coleman R.E. (2006). Magnesium Depletion in Patients Receiving Cisplatin-based Chemotherapy. Clin. Oncol..

[36] Li W.X., De Chen H., Wang X.W., Zhao S., Chen X.K., Zheng Y., Song Y. (2009). Predictive value of RIFLE classification on prognosis of critically ill patients with acute kidney injury treated with continuous renal replacement therapy. Chin. Med. J..

[37] Bagshaw S.M., George C., Dinu I., Bellomo R. (2008). A multi-centre evaluation of the RIFLE criteria for early acute kidney injury in critically ill patients. Nephrol. Dial. Transplant..

[38] Levey A.S., Inker L.A. (2017). Assessment of Glomerular Filtration Rate in Health and Disease: A State of the Art Review. Clin. Pharmacol. Ther..

[39] Murray P.T., Mehta R.L., Shaw A., Ronco C., Endre Z., Kellum J.A., Chawla L.S., Cruz D., Ince C., Okusa M.D. (2014). Potential use of biomarkers in acute kidney injury: Report and summary of recommendations from the 10th Acute Dialysis Quality Initiative consensus conference. Kidney Int..

[40] Pavkovic M., Robinson-Cohen C., Chua A.S., Nicoara O., Cárdenas-González M., Bijol V., Ramachandran K., Hampson L., Pirmohamed M., Antoine D.J. (2016). Detection of drug-induced acute kidney injury in humans using urinary KIM-1, miR-21, -200c, and -423. Toxicol. Sci..

[41] Sabbisetti V.S., Waikar S.S., Antoine D.J., Smiles A., Wang C., Ravisankar A., Ito K., Sharma S., Ramadesikan S., Lee M. (2014). Blood kidney injury molecule-1 is a biomarker of acute and chronic kidney injury and predicts progression to ESRD in type I diabetes. J. Am. Soc. Nephrol..

[42] Karasawa T., Steyger P.S. (2015). An integrated view of cisplatin-induced nephrotoxicity and ototoxicity. Toxicol. Lett..

[43] Hartmann J.T., Lipp H.P. (2003). Toxicity of platinum compounds. Expert Opin. Pharmacother..

[44] Tang K.W.A., Toh Q.C., Teo B.W. (2015). Normalisation of urinary biomarkers to creatinine for clinical practice and research—When and why. Singap. Med. J..

[45] Waikar S.S., Sabbisetti V.S., Bonventre J.V. (2010). Normalization of urinary biomarkers to creatinine during changes in glomerular filtration rate. Kidney Int..

[46] Moralidis E., Papanastasiou E., Didangelos T., Hilidis I., Siountas A., Arsos G. (2020). Determination of the glomerular filtration rate in patients with type 2 diabetes: An assessment of the agreement between 51Cr-EDTA plasma clearance and 99mTc-DTPA plasma clearance, 99mTc-DTPA renography and plasma creatinine prediction equation. Diabetes Res. Clin. Pract..

[47] Gabriel I.C., Nishida S.K., Kirsztajn G.M. (2011). Cistatina C sérica: Uma alternativa prática para avaliação de função renal?. Braz. J. Nephrol..

[48] Schley G., Köberle C., Manuilova E., Rutz S., Forster C., Weyand M., Formentini I., Kientsch-Engel R., Eckardt K.U., Willam C. (2015). Comparison of plasma and urine biomarker performance in acute kidney injury. PLoS ONE.

[49] Ishitsuka R., Miyazaki J., Ichioka D., Inoue T., Kageyama S., Sugimoto M., Mitsuzuka K., Matsui Y., Shiraishi Y., Kinoshita H. (2017). Impact of acute kidney injury defined by CTCAE v4.0 during first course of cisplatin-based chemotherapy on treatment outcomes in advanced urothelial cancer patients. Clin. Exp. Nephrol..

[50] Hashim D., Genden E., Posner M., Hashibe M., Boffetta P. (2019). Head and neck cancer prevention: From primary prevention to impact of clinicians on reducing burden. Ann. Oncol..

[51] Cai J., Jiao X., Luo W., Chen J., Xu X., Fang Y., Ding X., Yu X. (2019). Kidney injury molecule-1 expression predicts structural damage and outcome in histological acute tubular injury. Ren. Fail..

[52] Schag C.C., Heinrich R.L., Ganz P.A. (1984). Karnofsky performance status revisited: Reliability, validity, and guidelines. J. Clin. Oncol..

[53] Perazella M.A. (2018). Pharmacology behind common drug nephrotoxicities. Clin. J. Am. Soc. Nephrol..

[54] NCI, NIH (2009). Common Terminology Criteria for Adverse Events Version 4.03.

[55] Cockcroft D.W., Gault M.H. (1976). Prediction of creatinine clearance from serum creatinine. Nephron.

[56] Levey A.S., Bosch J.P., Lewis J.B., Greene T. (1999). A More Accurate Method To Estimate Glomerular Filtration Rate from Serum Creatinine: A New Prediction Equation. Ann. Intern. Med..

[57] Levey A.S., Stevens L.A., Schmid C.H., Zhang Y., Castro A.F., Feldman H.I., Kusek J.W., Eggers P., Van Lente F., Greene T. (2009). A new equation to estimate glomerular filtration rate. Ann. Intern. Med..

